# Deuteration-Induced Volume Phase Transition Temperature Shift of PNIPMAM Microgels

**DOI:** 10.3390/polym11040620

**Published:** 2019-04-03

**Authors:** Marian Cors, Lars Wiehemeier, Julian Oberdisse, Thomas Hellweg

**Affiliations:** 1 Department of Physical and Biophysical Chemistry, Bielefeld University, Universitätsstr. 25, 33615 Bielefeld, Germany; marian.cors@uni-bielefeld.de (M.C.); lars.wiehemeier@uni-bielefeld.de (L.W.); 2 Laboratoire Charles Coulomb (L2C), University of Montpellier, CNRS, 34095 Montpellier, France

**Keywords:** microgel, deuteration, isotope effect, NIPMAM, VPTT shift

## Abstract

The effect of deuteration on the volume phase transition (VPT) temperature of poly (*N*-isopropylmethacrylamide) (pNIPMAM) microgels in aqueous suspension is determined via IR spectroscopy and size measurements by photon correlation spectroscopy (PCS). We study the effect of a hydrogenated and a deuterated solvent (H_2_O/D_2_O), and of the hydrogenated and (partially) deuterated monomer. Deuteration of the monomer or copolymerization with deuterated monomers shifts the volume phase transition temperature (VPTT) by up to 8.4 K to higher temperatures, in good agreement with known results for pNIPAM microgels. Moreover, the shape of the swelling curve is found to depend on deuteration, with the highest deuteration leading to the sharpest VPT. Finally, the quantitative agreement between FTIR spectroscopy and PCS evidences the spatial homogeneity of the microgel particles. Our results are rationalized in terms of the effect of deuteration on hydrogen bonding. They shall be of primary importance for any experimental measurements close to the VPT involving isotopic substitution, and in particular contrast variation small angle neutron scattering.

## 1. Introduction

Acrylamide-based microgels are colloidal gels in the size range of some 10 nm to one micrometer [1]. The particle structure consists of a cross-linked polymer network of acrylamides, usually swollen by water. These kind of microgels show reversible shrinking and swelling as a function of external stimuli, for example temperature [1,2]. This property makes them suitable in various kinds of applications like drug delivery [3,4,5,6,7], photonic crystals [8] and photonic structures [9], micro and nanoreactors [10,11,12,13], smart surface coatings [14,15], and sensors [16,17]. At a specific temperature, the so-called volume phase transition temperature (VPTT), the microgel changes its size. The most prominent and studied microgels are poly (*N*-isopropylacrylamide) (pNIPAM) based microgels. The VPT of these microgels is approximately 32 °C [18,19,20,21]. Other prominent examples are poly (*N*-isopropylmethacrylamide) (pNIPMAM) with a VPTT of approximately 44 °C [20,21,22,23,24] and poly (*N*-*n*-propylacrylamide) (pNNPAM) with a VPTT of approximately 21 °C [23]. In the results section, we will show the hydrodynamic radius *R*_H_ as a function of the temperature measured with photon correlation spectroscopy (PCS) to illustrate the above-mentioned and well-known different volume phase transition for the three monomers. Note that the different amounts of used cross-linker *N*,*N*-methylenebisacrylamide (BIS) do not shift the VPTT, but may broaden the phase transition [25].

The VPTT of microgels based on these acrylamides are well-studied. There are also investigations of co-polymers combining two monomers in one microgel. Iwai et al. studied the VPTT of pNIPAM-co-pNIPMAM and pNNPAM-co-pNIPAM microgels with fluorescence spectroscopy [26]. Wedel et al. did turbidity and dynamic light scattering measurements on pNNPAM-co-pNIPMAM microgels and found that the VPTT shifts linearly with the copolymerization ratio [23]. Besides the VPTT, the geometry of homo-polymer [27,28,29,30], co-polymer [31,32], and functionalized [33] microgels, along with more complex architectures, such as core-shell [22,25,34,35,36,37,38], core-shell-shell [39,40], hollow-shell [41] or hollow-shell-shell [39,40,42] structures have also been investigated by small angle neutron scattering (SANS). One way to investigate the architecture of microgels with SANS is to use deuteration [30,43,44,45]. This approach is suitable for complex co-polymer microgels. One can match, for example, the deuterated monomer while investigating the shape of the non-deuterated microgel part, and vice versa. To assure that the shape of partially deuterated microgels represents the shape of non-deuterated microgels at a given temperature, it is important to know the influence of deuteration on the temperature-dependence of swelling. Furthermore, (partially) deuterated polymers are important for NMR studies for structural determination [46,47,48].

While the VPTTs of different acrylamide-based microgels are well known, the effect of deuteration on the VPTT has only been sparsely studied. In the literature, some early work from Crowther and coworkers on the effect of D_2_O as a solvent on the VPTT of pNIPAM is found and explained by a different strength in hydrogen bonding [49,50]. Berndt et al. [22] reported the same effect for pNIPAM and pNIPMAM, and Nöjd et al. [45] and Mohanty et al. [43] evidenced an effect of the deuteration of the monomer itself, but to the best of our knowledge there is no systematic study of the effect of deuteration of the polymer itself on the behavior of pNIPMAM-based microgels. In particular, pNIPMAM microgels with different levels of deuteration or deuterated or hydrogenated pNIPMAM copolymers need to be investigated, as such knowledge is crucial for SANS experiments. In this work, we explore the effect of hydrogenated (H), partially (D7) and fully (D12)-deuterated monomers, the effect of D7/H12-pNIPMAM copolymers, and hydrogenated and deuterated solvent (H_2_O/D_2_O) on the VPTT of pNIPMAM microgels.

## 2. Materials and Methods

### 2.1. Synthesis

**Scheme 1:**
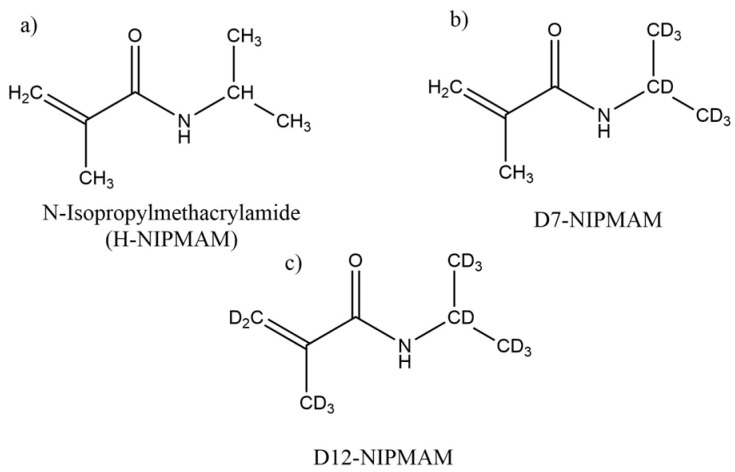
Chemical structure of (**a**) H-pNIPMAM, (**b**) D7-pNIPMAM, and (**c**) D12-pNIPMAM. The amide-H exchanges with the solvent.

The detailed syntheses of *N*-isopropylmethacrylamide (NIPMAM, Sigma-Aldrich, purity > 97%) microgels has been described elsewhere [15]. Here we also used D7-*N*-isopropylmethacrylamide (D7-NIPMAM, Polymer Source. Inc., Montreal, Quebec, Canada) and D12-*N*-isopropylmethacrylamide (D12-NIPMAM, Polymer Source. Inc., Montreal, Quebec, Canada) as the monomers. The syntheses of (partially) deuterated microgels were similar to the one with NIPMAM. For all syntheses, we performed a precipitation polymerization under nitrogen atmosphere in 50 mL water. The concentration of the monomer was always 0.082 mol·L^−1^. The cross-linker was *N*,*N*-methylenebisacrylamide (BIS, 99%, 0.0082 mol·L^−1^), the initiator was ammonium peroxodisulfate (>98%, 0.0027 mol·L^−1^), and the surfactant was sodium dodecyl sulfate (>99%, 0.0049 mol·L^−1^). All non-deuterated chemicals were purchased from Sigma-Aldrich (St. Louis, MO, USA). The different chemical structures of the (partially) deuterated monomers are shown in Scheme 1.

Furthermore, we synthesized co-polymer microgels of NIPMAM and D7-NIPMAM in different ratios. For the co-monomer synthesis, the two monomers were added at the same time. The deuterated/hydrogenated ratios were 0/100, 25/75, 50/50, 75/25, and 100/0.

### 2.2. Photon Correlation Spectroscopy

We used photon correlation spectroscopy (PCS) to study the diffusional properties of the microgels (angle dependent) and the change in size with temperature (temperature dependent). The temperature-dependent PCS measurements were performed with a partly home-made setup at a fixed angle of 60°. The set-up was built with a He-Ne laser (HNL210L-EC, 632.8 nm, Thorlabs, Newton, MA, USA) as the light source, a multiple tau digital correlator (ALV-247 6010, ALV GmbH, Langen, Germany), and a photodiode detector (SO-SIPD, ALV GmbH, Langen, Germany). For temperature control, the sample was placed in a decalin matching bath thermostated with a refrigerated bath (Haake C25P, Thermo Fisher Scientific, Waltham, MA, USA) equipped with a controller (Phoenix II, Thermo Fisher Scientific, Waltham, MA, USA).

The angle-dependent PCS measurements were done using a 3D light scattering spectrometer (3D LS Spectrometer, LS Instruments AG, Fribourg, Switzerland) equipped with a He-Ne laser (1145P, JDSU, Milpitas, CA, USA). The sample was placed in a decalin matching bath. The temperature was controlled with a refrigerated bath (JULABO F25, Julabo Labortechnik GmbH, Seelbach, Germany) equipped with a controller (JULABO ME, Julabo Labortechnik GmbH, Seelbach, Germany).

The PCS experiments yield the intensity time autocorrelation functions of the samples, which can be converted into the respective correlation function g^1^ (t) of the electrical field. g^1^ (t) is analyzed using the method of cumulants [51,52] leading to the averaged relaxation rate *Γ*. For purely diffusional dynamics, *Γ* is expected to follow:

(1) $$\Gamma =D\;q^{2}$$

With

(2) $$q=\;\frac{4\pi n}{\lambda }\;sin\left(\frac{\theta }{2}\right)$$

and with *D* being the Fickian diffusion coefficient. With the Stokes-Einstein equation, the Boltzmann constant *k*_B_, the temperature *T*, and the viscosity *η* of the hydrodynamic radius *R*_H_ of the particle can be calculated.

(3) $$R_{H}=\;\frac{k_{B}T}{6\pi \eta D}$$

### 2.3. Infrared Spectroscopy

We have recently described the procedure of Fourier transform infrared (FTIR) spectroscopy of microgels in a dedicated article [53]. In short, the used FTIR spectrometer (Tensor 27, Bruker, Billerica, MA, USA) was controlled with OPUS 7.5 (Bruker, Billerica, MA, USA). The spectrometer was equipped with a home-made brass sample holder, which was temperature controlled with a precision of 0.1 K with a refrigerated thermostat (model 1166D, VWR International, Radnor, PA, USA). During the measurements, the spectrometer was purged with dried air generated by a purge gas generator (model 75-62, Parker Hannifin, Cleveland, OH, USA). Concentrated microgel samples (1 µL) in D_2_O were pipetted onto a BaF_2_ (Korth Kristalle, Kiel, Germany) cuvette, ensuring an optical path length of 7 µm. Absorbance spectra were calculated from sample transmission spectra and temperature dependent spectra of D_2_O was acquired within a two-week period.

### 2.4. Atomic Force Microscopy

We used an atomic force microscope (Bruker, Billerica, MA, USA) to evidence sphericity and measure the size distribution of microgels. Measurements were performed with silicon tips (RTESP, Bruker, Billerica, MA, USA). The measurement was done in the tapping mode with a frequency of 318 kHz using a controller (NanoScope V, Veeco, Plainview, TX, USA). A droplet (3 µL) of a highly diluted (ca. 0.0001 wt %) aqueous microgel solution was put on a silicon wafer (Sigert Wafer, diameter = 50.8 mm, Aachen, Germany) and dried in a dust-free environment in air.

## 3. Results

### 3.1. Particle Size and Shape

As mentioned in the introduction, SANS is a suitable technique to study the size, shape and internal structure of microgels. Most of the models have the condition that the microgel is spherically symmetric. In the following, we characterized our pNIPMAM particles with angle-dependent PCS and atomic force microscopy (AFM) measurements.

**Figure 1:**
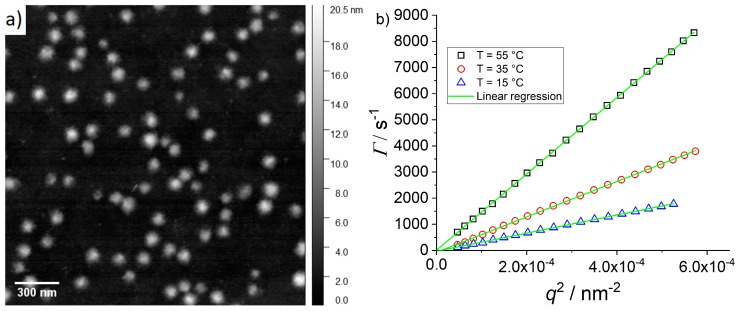
PCS and AFM measurements of a H-pNIPMAM microgel. (**a**) Atomic fore microscopy image of dried pNIPMAM microgels on a silicon substrate. (**b**) The *Γ* vs. *q*^2^ of an angle dependent PCS measurement in water from 30° to 130° at 15, 35, and 55 °C. The Y-Axis intercepts are −32, −75, and 5 s^−1^, respectively.

In the AFM image in Figure 1a (left), the pNIPMAM particles are symmetric in the two lateral dimensions. This suggests a spherical shape in suspension because anisotropic shaped particles would result in anisotropic projections if they are not preordered. Furthermore, as this measurement was done with dried microgels in air, the particles show (for spherical microgels typical) the rather flat pancake-like structure [15,33,54,55,56].

The graph in Figure 1b (right) shows the average relaxation rate *Γ* of the experimental correlation functions of the electrical field g^1^ (t) as a function of the squared magnitude of the scattering vector *q*^2^ for a H-pNIPMAM microgel at 15, 35, and 55 °C. For all three measurements, the *Γ*-axis intercept is zero within the error margins. As the intercept of the linear regression is zero within the experimental precision and the microgels following Equation (1), they show only translational diffusion, with *D* being the translational diffusion coefficient. Assuming a spherical shape with Equation (3), the diffusion coefficient can be converted into the hydrodynamic radius.

As these two measurements (AFM and PCS) are in good agreement, indicating a spherical symmetry, we determined the polydispersity in the radius of approximately 20% by the size distribution of the AFM image.

### 3.2. Swelling Behavior of Microgels

As discussed in the introduction, the volume phase transition (VPT) of acrylamide-based microgels is well known. This is particularly true for the shift of the VPTT of fully hydrogenated poly(*N*-*n*-propylacrylamide) (pNNPAM VPTT ≈ 22 °C) over poly(*N*-isopropylacrylamide) (pNIPAM, VPTT ≈ 32 °C) to poly(*N*-isopropylmethacrylamide) (pNIPMAM, VPTT ≈ 44 °C) in H_2_O.

**Figure 2:**
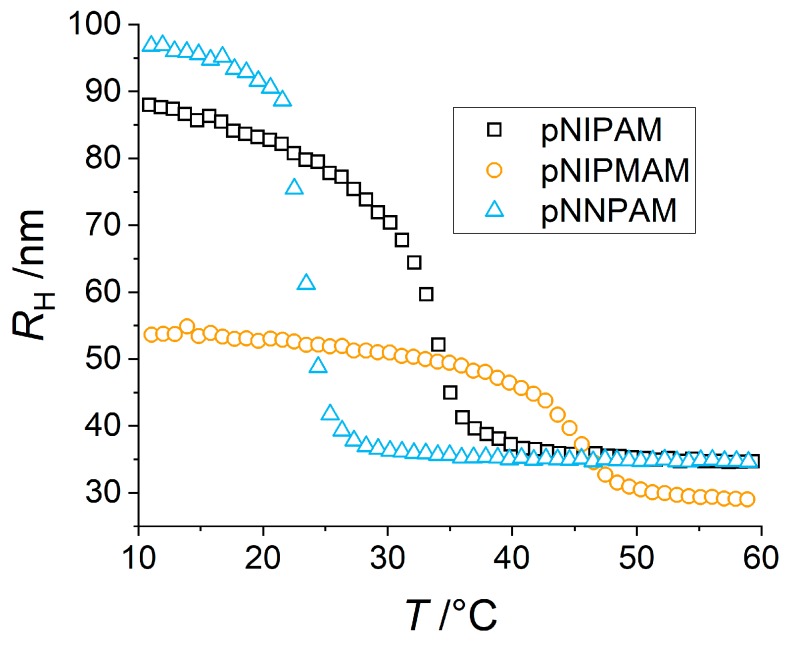
Hydrodynamic radius of pNNPAM (1.9 mol% BIS), pNIPAM (5.0 mol% BIS), and pNIPMAM (10.0 mol% BIS) as a function of the temperature. The curves were measured in water for particles synthesized under identical conditions.

In order to exemplarily define our approach applied to deuterated microgels or solvents, Figure 2 shows the change in size (hydrodynamic radius) with the temperature for these three microgel species. At low temperatures (here 10 °C) the microgel is swollen by the solvent (water). This swollen state of an acrylamide-based microgel is stabilized by the hydrogen-bonding between the polymer and the solvent (water). By an increase in temperature the solvent-polymer hydrogen-bonds become less preferred compared to intermolecular interactions, and the polymer releases the water, causing the microgel to collapse. Thus, at high temperatures the microgel is in the collapsed state. The temperature where the collapse takes place is the VPTT and is usually defined as the inflection point of the hydrodynamic radius curve as a function of the temperature. The difference in structure of the above mentioned acrylamide monomers, and in particular (partial) deuteration, affect the polymer-solvent hydrogen bonds and result in different VPTT. In order to highlight this shift in the VPTT, we normalized the hydrodynamic radius as a function of the temperature as follows:

(4) $$S=\frac{R_{H}\left(T\right)-R_{H}\left(min\right)}{R_{H}\left(max\right)-R_{H}\left(min\right)}$$

**Figure 3:**
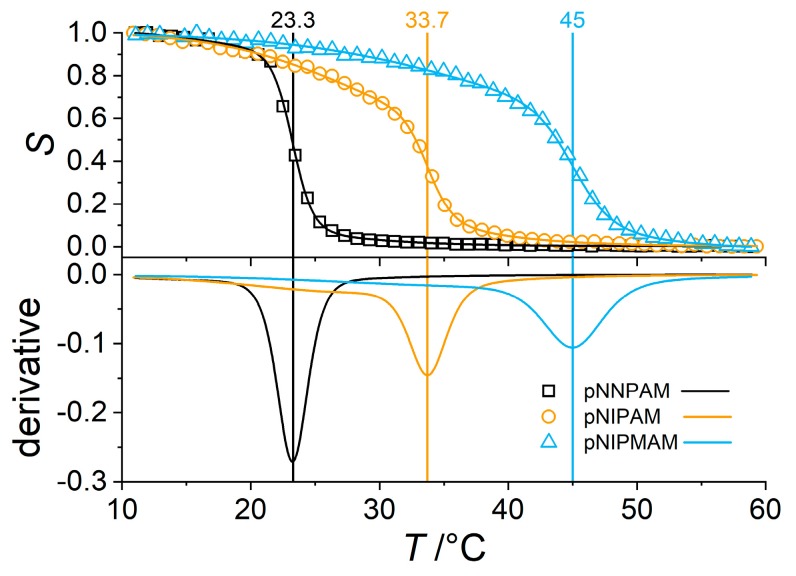
(**Top**) Normalized swelling curves of the size ranging from 0 to 1 of pNNPAM (1.9 mol% BIS), pNIPAM (5.0 mol% BIS), and pNIPMAM (10.0 mol% BIS) as a function of the temperature. The curves where measured in water for particles synthesized under identical conditions. (**Bottom**) Derivative of the fit describing the experimental data in the upper part of the figure. The vertical lines indicate the VPTT.

Using Equation (4) results in a normalization of the hydrodynamic radius in the range from 0 to 1. The normalized swelling curves of pNNPAM, pNIPAM, and pNIPMAM are shown in the upper part of Figure 3.

To determine the VPTT we described the experimental data of the normalized hydrodynamic radius as a function of the temperature with a sigmoidal fit (see SI for details). We then calculated the derivative of this fit. In the lower part of Figure 3, the derivative as a function of the temperature is shown for the fit in the upper part of the Figure. The vertical lines indicate the inflection point, thus the VPTT of each microgel respectively. In the following sections we will determine the VPTT of hydrogenated and deuterated pNIPMAM microgels as well as pNIPMAM microgels in light and heavy water in the same way. Furthermore, the broadness of the volume transition can be quantified by the full width at half maximum (FWHM) of the derivative. Djokpé et al. found very similar shapes of the phase transition of linear pNIPAM and pNIPMAM particles. Here the broadness of each microgels is different. For pNIPAM microgels, Kratz et al. found an increase in the volume transition broadness with an increase in cross-linker content [57]. Moreover, they found an increase in the swelling ratio of the collapsed and swollen state with a decrease in cross-linker content. This could be due to an increase in the fuzziness of the particle, as this would increase the hydrodynamic radius mainly in the swollen state. Wu et al. compared the “coil-to-globule transition” of linear chains with the “volume phase transition” of microgels [58]. Based on these findings, we will discuss the difference in volume transition broadness in each respective section.

### 3.3. Effect of PNIPMAM Monomer Deuteration

Although the VPT of such hydrogenated microgels is well studied, the VPT of their (partially) deuterated homologues remains mostly unknown. Here, we present the swelling curves of pNIPMAM with three different levels of deuteration. The fully hydrogenated monomer H-pNIPMAM (Scheme 1a in experimental section), the partially deuterated monomer D7-pNIPMAM (Scheme 1b) where the isopropyl group is deuterated, and the fully deuterated D12-pNIPMAM (Scheme 1c). Note that the nitrogen bound hydrogen is in exchange with the solvent.

**Figure 4:**
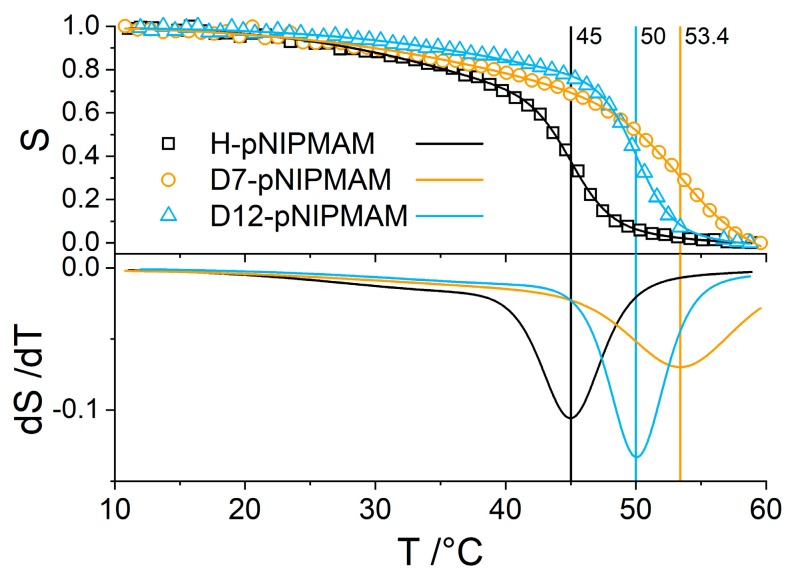
(**Top**) Swelling curves of pNIPMAM microgels in water with different levels of deuteration of the monomers. (**Bottom**) Derivative of the fit of the experimental data in the upper part. The vertical lines at the peaks indicate the VPTT of the respective microgel.

At first we studied the influence of (partial) deuteration of the NIPMAM monomer. The upper part of Figure 4 shows the normalized hydrodynamic radius as a function of the temperature. The lower part of Figure 4 shows the derivative. The VPTT is indicated by vertical bars.

We determined the VPTT of H-pNIPMAM to be 45.0 °C. This is in very good agreement with the literature [20,21,22,23,24,59]. The VPTT of the partially deuterated D7-pNIPMAM is at 53.4 °C and the one of fully deuterated D12-pNIPMAM is 50.0 °C. Comparing H-pNIPMAM with partially deuterated D7-pNIPMAM shows that the deuteration of the isopropyl group shifts the VPTT by 8.4 K towards higher temperatures. A deuteration of the isopropyl group of the monomer results in deuteration of the sidechains in the microgel polymer network. In a previous study, a shift of 5 K by deuteration of the isopropyl group of microgels made with a different monomer (D7-pNIPAM) was explained by the isotope effect [45]. As mentioned above, the VPT depends on the polymer-solvent hydrogen bonds. The β-deuteration of amines, here the deuteration of the isopropyl group, increases their basicity [60]. Furthermore, it is known that microgels interact via intermolecular and intramolecular hydrogen bonds [61]. Both the basicity and the hydrogen bonds are based on the difference in electronegativity and are affected by a change in electron density at the C=O and the N–H group by a β-deuteration. Thus under deuteration, the polymer-polymer and solvent-solvent interaction becomes less favorable than solvent-polymer interactions, increasing the VPTT. Furthermore, hydrophobic interactions are known to be weakened by deuteration [62] and the weakened intramolecular hydrophobic interactions shift the VPTT towards higher temperatures [63]. Thus these two effects can explain the relatively large shift in VPTT. Compared to the hydrogenated microgel, a full deuteration (D12) of the monomer results in a shift of only 5 K towards high temperatures; thus, 3.4 K less compared to the D7 case. Here, for the fully deuterated (D12) microgel, both the sidechains and the backbone of the polymer are deuterated. As the phase transition of a microgel is based on the balance between polymer-polymer and polymer-solvent interactions, the intramolecular interactions in a fully deuterated microgel seem to disrupt the polymer-solvent H-bond network more efficiently than a partially deuterated polymer. This is analogous to the difference between pNIPAM and pNIPMAM, where the absence of the methyl group induces a lower VPTT [64]. A deuteration of the methyl group reduces the C–H/D bond length [65], and thus the sterical hindering of the orientation of the hydrophobic groups. Beside the shift in VPTT, also the shape of the size transition changes. This can already be seen in the upper part of Figure 4. The shape of the transition of the fully deuterated microgel is sharper and steeper than the shape of the hydrogenated microgels, and the shape of the VPT of the partially deuterated microgel is much broader and has a lower steepness. This difference in shape is even more visible from the peak broadness and magnitude of the derivative in the lower part of Figure 4. To quantify the broadness of the VPT, we have calculated the broadness of the peak of the derivative in temperature by the FWHM, and call this the broadness of the VPT. For the hydrogenated microgel, it is 6 K. The partially deuterated microgel has a much broader VPT at 11.4 K, and the VPT of the D12 microgel is sharpest at 5 K. As discussed above, this change in broadness could indicate a lower fuzziness at the particle interface of the D12 microgel.

In summary, by a deuteration of only the isopropyl group of NIPMAM, the VPTT is shifted more than if the whole monomer were deuterated. The shape of the VPT depends on deuteration; it is broadened for the partially deuterated D7-pNIPMAM and sharpened for the fully deuterated D12-pNIPMAM. Thus, one of the key findings of this work is that the VPTT changes with the degree of deuteration of the monomer, and furthermore, the shape of the VPT curve is modified as well.

### 3.4. Partially Deuterated Co-Polymers

**Figure 5:**
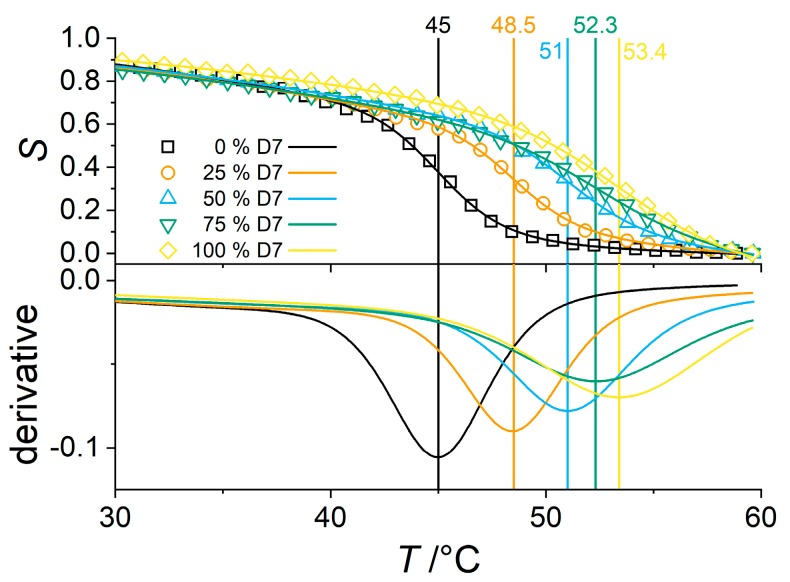
(**Top**) VPTT of four D7/H-pNIPMAM co-polymer microgels in water with different co-polymer ratios. The percentage of H-pNIPMAM is indicated in the legend. (**Bottom**) Derivative of the fit of the experimental data in the upper part. The vertical lines at the peaks indicate the VPTT of each microgel.

After studying the effect of different levels of deuteration of the monomers, we synthesized co-polymer microgels of H-pNIPMAM and D7-pNIPMAM. We choose H- and D7-pNIPMAM for the co-polymer microgels because the difference in the VPTT is the largest for these two species. In addition to the already studied homo-polymer particles (0 mol% D7 and 100 mol% D7), we decided to analyze three further intermediate ratios of H-pNIPMAM and D7-pNIPMAM co-polymer microgels (25, 50, and 75 mol% D7). The upper part of Figure 5 shows the normalized size transition with the temperature for H-pNIPMAM and D7-pNIPMAM co-polymer microgels with the co-polymer content of D7-pNIPMAM indicated in the legend. The lower part shows the derivative of a fit representing the experimental data in the upper part of the figure. Again the peak position of the derivative is the VPTT. It is indicated by the vertical straight lines labelled by the respective VPTT in °C.

Increasing the rate of deuteration of the co-polymer microgels shifts the VPTT from 45 °C for H-pNIPMAM over 48.5 °C (25% D7-pNIPMAM), 51 °C (50% D7-pNIPMAM), and 52.3 °C (75% D7-pNIPMAM), to 53.4 °C for the D7-pNIPMAM. Thus, a step of 3.5 K by an exchange of the first 25% of monomer, a further step of 2.5 K for the next 25%, followed by one of 1.3 K, and finally 1.1 K for the last 25%. This means that the VPTT of pNIPMAM-based microgels can be tuned by the degree of co-polymerization of deuterated and non-deuterated monomers. Furthermore, as indicated by the successive steps, the shift of the VPTT with the degree of the co-polymer ratio is non-linear and seems to saturate progressively (see Figure 7 and the discussion below). This is in contrast to co-polymers made with different types of monomers [23]. Moreover, the shape of the VPT changes with the co-polymerization ratio. The broadness almost doubles from 6 K for the co-polymer with 0% D-pNIPMAM over 6.4 K (25% D7), 7.8 K (50% D7), 11.8 K (75% D7), to finally 11.4 K for the D7-pNIPMAM homo-polymer (100% D7). These two results hint towards a complex influence of the deuteration on the hydrogen-bonding within the polymer network.

With the exception of the co-polymer with a D7-pNIPMAM content of 75%, the VPT is broadened from the pure hydrogenated microgel over the co-polymer microgels to the deuterated microgel. Given that the shift in VPTT saturates, one might assume the same for the broadness, which can be seen as identical within error bars above 75% of deuteration. The general shape of the three co-polymers is similar to the one of the two homo-polymers. The co-polymers show one VPT. It is known in literature that microgels with large domains of different polymers show several features in the VPT, such as two steps as core-shell microgels [34]. The fact that our microgels do not show a two-step VPT or a kink in the swelling curve is a hint that the H/D7 ratio is homogeneous in the whole microgel. Thus, the two monomers, D7-NIPMAM and H-NIPMAM, seem to have approximately the same reaction velocity, as very different reaction velocities would result in larger H- and D7-rich domains, even though the difference in the volume transition broadness indicates a difference in the fuzziness.

Interestingly, the fully deuterated homo-polymer microgel also exhibits a broadened phase transition compared to the hydrogenated specimen. Further, the phase transition temperature depends on the degree of deuteration in a non-linear way. These two results hint towards a complex influence of the deuteration on the hydrogen-bonding within the polymer network, but there seem to be no larger homo-polymer domains with an effect on the temperature-dependent swelling curve.

Here, we detected only the hydrodynamic radius of the whole particle as a function of the temperature by PCS. In the following we used FTIR-spectroscopy to investigate if there are inhomogeneities inside the particle which do not affect the VPT as characterized by the global size of the particle. In a previous study we applied temperature dependent FTIR-spectroscopy of the NH-band as a direct probe for the hydrogen bonding. Moreover, we established IR-spectroscopy to study co-polymer microgels [53]. In this work, we aim to apply FTIR spectroscopy of microgels in D_2_O [66,67,68] to ensure that the hydrogenated and deuterated monomers copolymerize in a statistical manner on the molecular level. The strength of FTIR spectroscopy is the probing of the entire particles, regardless of the location of the monomer inside the microgels. This allows gathering of information on the particle architecture. As shown before [53], a statistical co-polymerization leads to only one phase transition in FTIR, whereas larger homo-polymer domains and gradients in composition result in separated phase transitions or a broadened phase transition compared to the homo-polymers, respectively.

**Figure 6:**
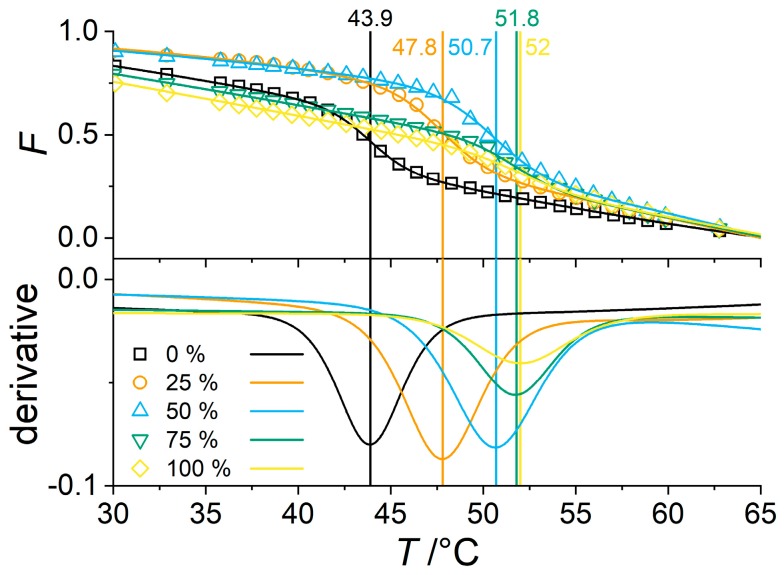
(**Top**) Dependence of the normalized wavenumber of the N-D-vibration on the temperature for five D7-pNIPMAM-H-pNIPMAM co-polymers with 0, 25, 50, 75, and 100% D7-pNIPMAM measured by FTIR spectroscopy in heavy water. A representative temperature-dependent FTIR spectroscopy measurement is shown in the SI in Figure S4. (**Bottom**) Derivative of the fit in the upper part of the graph. The vertical lines indicate the VPTT.

In contrast to past work, here we performed measurements in D_2_O, because in the resulting spectra there is no overlap of the d (OH) vibration from H_2_O with the ones of the microgels. This enables an easier acquisition of the spectra, as no strict control over the sample thickness is necessary, allowing for background correction. However, this modification leads to the exchange of the amide H with D from the solvent, and we thus study here the d (ND) vibration at about 1450 cm^−1^ in spite of the d (NH) vibration. As we are interested in the VPTT, we normalized the wavenumber of the N–D vibration to the range of 0–1 in the same way as the hydrodynamic radius before (see SI for details). The upper part of Figure 6 shows the normalized wavenumber *F* of the N–D vibration as a function of the temperature and the lower part as the derivative of the fit.

**Figure 7:**
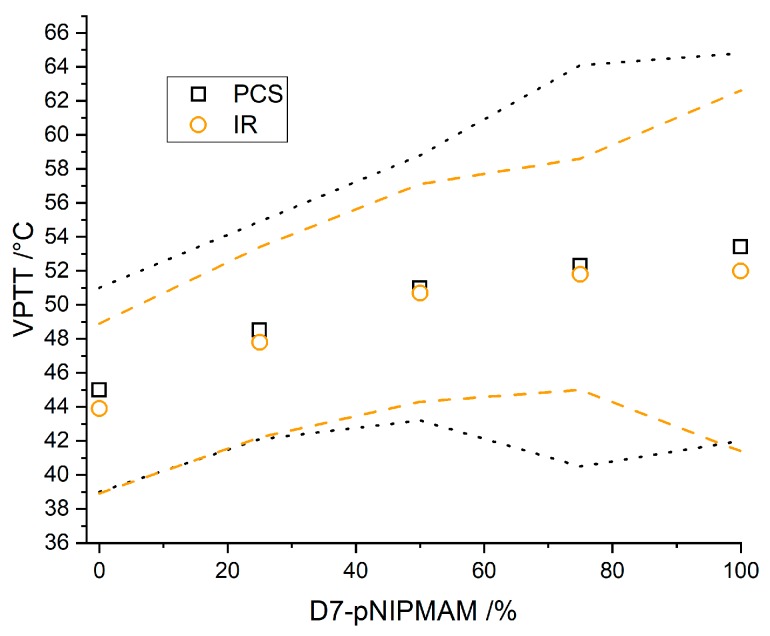
VPTTs (symbols) of D7/H-pNIPMAM co-polymers measured by PCS in water and IR-spectroscopy in heavy water as a function of the D7-NIPMAM content. The broadness of the VPT is indicated via the upper and lower limit of the transition represented with dashed (IR) and dotted (PCS) lines.

The vertical line at the minimum of the derivative in the lower part of Figure 6 indicates the VPTT. It follows the same trend as in the PCS measurements. For the hydrogenated microgel, the VPTT is at 43.9 °C for the co-polymer with 25% D7 at 47.8 °C, for 50% D7 at 50.7 °C, for 75% D7 at 51.8 °C, and for the fully deuterated microgel at 52.0 °C. In Figure 7 the VPTT is plotted as a function of the D7-NIPMAM content for PCS and FTIR-spectroscopy measurements. The findings of both methods are in good agreement. The slight shift of 0.3–1.4 K of the FTIR measurements compared to the PCS measurement can be explained by the change of solvent from H_2_O to D_2_O, as will be discussed below. Also, for the broadness of the VPT we have the same findings for IR-spectroscopy as for PCS. For IR-spectroscopy the broadness of the VPT is 5.0 K for the H-pNIPMAM microgel, 5.6 K for the co-polymer with 25% D7, 6.4 K for 50% D7, 6.8 K for 75% D7, and 10.8 K for 100% D7 (Figure 7). Even more interestingly, also with FTIR-spectroscopy we can only detect a single phase transition. This result indicates a successful statistical copolymerization of both hydrogenated and deuterated monomers throughout the whole particle. No core-shell architecture or gradient are present, as these would result in separate phase transitions or a further broadening of the phase transition.

One of the main outcomes of this work is thus that neither by PCS (particle size) nor by FTIR-spectroscopy (internal structure) domains with pure hydrogenated or deuterated species of the polymer could be detected. Both techniques hint to a microgel with an approximately constant deuterated/hydrogenated polymer throughout the microgel. Furthermore, the VPTT is shifted in the range of the two homo-polymers, and the broadness of the transition is increased with the D7/H ratio.

### 3.5. Influence of the Solvent on the VPTT of PNIPMAM Microgels

**Figure 8:**
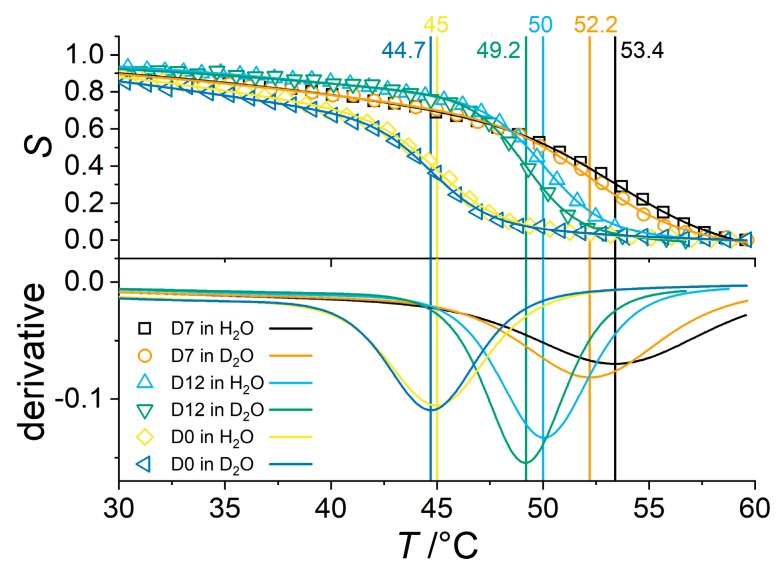
Influence of the solvent (H_2_O/D_2_O) on the VPTT of H-, D7-, and D12-pNIPMAM. (**Top**) Normalized hydrodynamic radius vs. the temperature with a fit describing the experimental data. (**Bottom**) Derivative of the fit in the upper part of the figure with vertical lines indicating the VPTTs.

After studying the effect of different levels of deuteration of the monomer, in this subsection we analyze the isotope effect of the solvent (H_2_O/D_2_O). There are slight differences in the determined VPTT for PCS and FTIR. In D_2_O the VPTTs of H-pNIPMAM are 43.9 °C (FTIR) and 44.7 °C (PCS), and for D7-pNIPMAM are 52.0 °C (FTIR) and 52.2 °C (PCS), respectively (Figure 6 and Figure 8). This difference is due to the different measurement principles for PCS and FTIR. For the investigation of the difference in VPTT by changing the solvent, we therefore focused on PCS measurements. Thus, PCS measurements in light and heavy water have been performed. Note that the resulting diffusion coefficient is viscosity-corrected for H_2_O and D_2_O, respectively. In Figure 8 we display again the normalized size as a function of the temperature in the upper part and the derivative of the fits of the experimental data in the lower part.

First of all, the effect in terms of VPTT shift is minor when changing the solvent as compared to modifying the monomers. For all three samples the VPTT decreases when the solvent is changed from H_2_O to D_2_O. For D7-pNIPMAM, the VPTT decreases by 1.2 K, for D12-pNIPMAM by 0.8 K, and for H-pNIPMAM by 0.3 K. Thus, while the (partial) deuteration of the monomer itself and of H/D7 co-polymers shifts the VPTT towards higher temperatures, the deuteration of the solvent shifts the VPTT to lower temperatures. This can be attributed to the strength of the hydrogen bonding. As the phase transition temperature is dependent on both the polymer-polymer interaction and the polymer-solvent interaction, the influence of the hydrogen bonding from deuteration of both polymer and solvent can alter the VPTT. As the hydrogen bonding via deuterium is generally weaker than via protium [69,70,71,72], deuteration of the solvent leads to a decrease of the VPTT. This effect increases with the level of deuteration from H12 over D7 to D12 pNIPMAM.

## 4. Discussion

In this article we present a study of the deuteration-induced changes of the swelling behavior of pNIPMAM-based microgels. This work covers the influence of the use of heavy water as well as changes stemming from the use of partially of fully deuterated monomers. We found that a partial and full deuteration of the monomer shifts the VPTT by 8.4 K and 5 K to higher temperatures. This shift can be explained by a change in the strength of the hydrogen bonds and is in good agreement with findings for pNIPAM microgels in literature [43,45]. Co-polymerization of hydrogenated (H12-pNIPMAM) and partially deuterated (D7-pNIPMAM) also shifts the VPTT to higher temperatures with an increase in the ratio of the deuterated species, from 45 °C to approximately 53 °C. Furthermore, co-polymer microgels made of monomers with different levels of deuteration show a different shape of the VPT. It is broader for co-polymers with higher ratios of D7-pNIPMAM. Concerning the level of deuteration of the monomer, D12-pNIPMAM has the sharpest VPT while D7-pNIPMAM has the broadest VPT. These properties have been observed with PCS and FTIR-spectroscopy. Both techniques reveal similar values for the shift in VPTT and the broadness of the VPT. Another finding is the shift in the VPTT by changing the solvent from water to heavy water. Here the magnitude of the shift depends on the level of deuteration of the monomer and is always to lower temperatures. The highest shift was found for the fully deuterated D12-pNIPMAM (1.4 K) and the lowest for the hydrogenated H12-pNIPMAM (0.3 K). Thus, a much lower shift is evidenced for solvent changes than for the deuteration of the microgel itself. To the best of our knowledge, only findings for hydrogenated pNIPAM an pNIPMAM microgels showing a slight shift to higher temperatures have been reported in the literature [22,50]. Our results are thought to be highly relevant for studies of the microgel structure and geometry near the VPTT, such as SANS measurement, where deuteration is crucial for highlighting different parts of microgels using isotopic contrast variations or for NMR studies of such systems.

## Supplementary Materials

The following are available online at https://www.mdpi.com/2073-4360/11/4/620/s1, Figure S1: Left: hydrodynamic radius as a function of the temperate for H-pNIPMAM, D7-pNIPMAM, and D12-pNIPMAM. Right: Hydrodynamic radius normalized to 0–1 as mentioned in the main article. The measurement at 13 °C for H-pNIPMAM and at 55 °C for D12-pNIPMAM failed. Figure S2: FTIR-spectroscopy measurement of H/D7-pNIPMAM co-polymer microgels with the D7 content indicated in the legend. Left: Wavenumber of the d (ND) vibration as a function of the temperature. Right: Normalized wavenumber (with Equation S2) as a function of the temperature. The experimental data were fitted with Equation S1. Figure S1: Γ q^−2^ vs. q^2^ of an angle dependent PCS measurement in water from 30° to 130° at 15, 35 and 55 °C. Figure S4: FTIR measurement for a temperature range from 15.65 to 65.70 °C for a H-pNIPMAM microgel. The NH-band is highlighted in grey.
