# Supplementary material for: Deuteration-Induced Volume Phase Transition Temperature Shift of PNIPMAM Microgels
Source: Polymers (Basel). 2019 Apr 3;11(4):620. doi: 10.3390/polym11040620 (PMC6523740; doi:10.3390/polym11040620)
Supplement: Supplementary file 1 [file polymers-11-00620-s001.pdf]

polymers

MDPI

Supporting Information

Deuteration-Induced Volume Phase Transition Temperature shift of PNIPMAM microgels

Marian Cors [1,2], Lars Wiehemeier [1], Julian Oberdisse [2,*] and Thomas Hellweg [1,*]

[1] Department of Physical and Biophysical Chemistry, Bielefeld University, Universitätsstr. 25, 33615 Bielefeld, Germany

[2] Laboratoire Charles Coulomb (L2C), University of Montpellier, CNRS, 34095 Montpellier, France.

* Correspondence: JO: julian.oberdisse@umontpellier.fr, thomas.hellweg@uni-bielefeld.de

Received: date; Accepted: date; Published: date

Size Normalization

As mentioned in the article, we normalized the hydrodynamic radius of the different microgels to a scale from 1 to 0. All measurements of the hydrodynamic radius as a function of the temperature where done in the temperature range from 10 °C to 60 °C in 1 K steps. The highest value of R_H was usually obtained for 10 °C and the lowest value was usually obtained for 60 °C.

The left part of Figure S1 shows the hydrodynamic radius as a function of the temperature for three microgels with different levels of deuteration of the used NIPMAM monomer. The right part of Figure S1 shows the fit. We used the sigmoidal BiDoseResp fit with the program OriginLab.

$$y = A1 + (A2 - A1)\left[\frac{p}{(1+10^{(LOGx01-x)h1})} + \frac{1-p}{(1+10^{(LOGx02-x)h2})}\right] \quad \text{(S1)}$$

Here, A1 and A2 are the two plateaus before and after the two sigmoidal steps, h1 and h2 are the slopes at the inflection points of the two sigmoidal steps and p is a proportional factor.

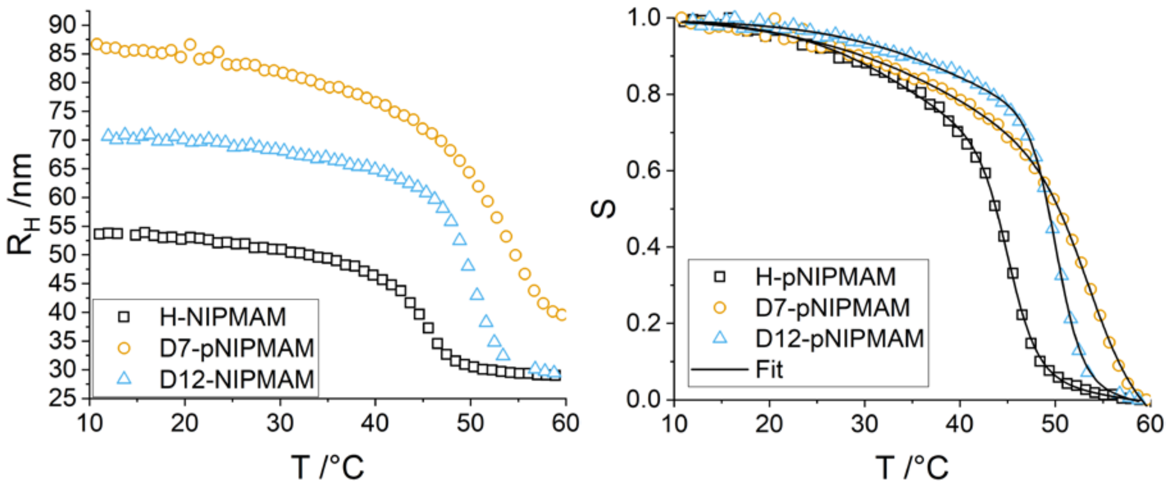

Figure S1. Left: hydrodynamic radius as a function of the temperate for H-pNIPMAM, D7-pNIPMAM and D12-pNIPMAM. **Right:** Hydrodynamic radius normalized to 0-1 as mentioned in the main article. The measurement at 13 °C for H-pNIPMAM and at 55 °C for D12-pNIPMAM failed.

Normalization of the wavenumber of FTIR-spectroscopy measurements

The wavenumber $\tilde{\nu}$ of the FTIR-spectroscopy measurements were normalized analogous to the size of the particles.

$$F = \frac{\tilde{\nu}(T) - \tilde{\nu}(min)}{\tilde{\nu}(max) - \tilde{\nu}(min)} \quad \text{(S2)}$$

On the left in Figure S2 the measured peak positions of the d(ND) vibration are plotted as a function of the temperature. The graph on the right shows the same data with the normalization of the wavenumber with eq. S2. The normalized data were fitted with ex. S1.

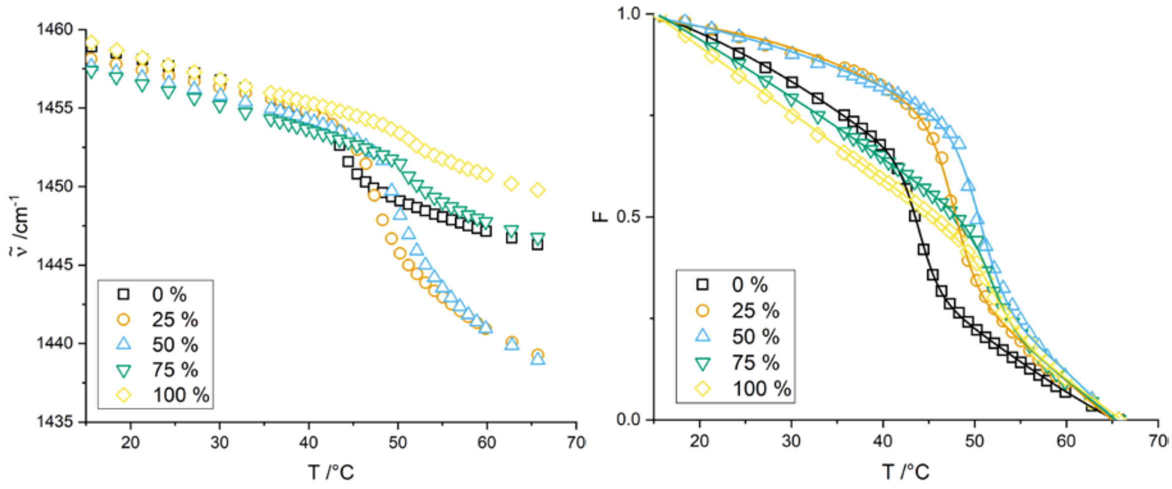

Figure S2. FTIR-spectroscopy measurement of H/D7-pNIPMAM co-polymer microgels with the D7 content indicated in the legend. **Left:** Wavenumber of the d(ND) vibration as a function of the temperature. **Right:** Normalized wavenumber (with Eq. S2) as a function of the temperature. The experimental data were fitted with eq. S1.

Change in diffusion coefficient

To determine if the diffusions coefficient ($D = \Gamma\, q^{-2}$) is constant for all angles, $\Gamma\, q^{-2}$ was plotted as a function of q^{-2} in Figure S3.

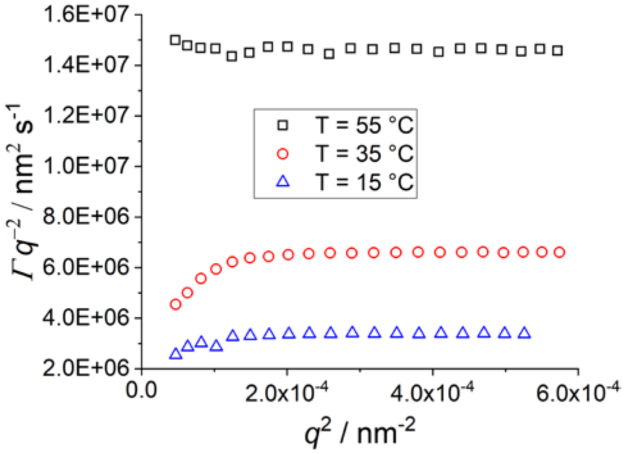

Figure S 3: $\Gamma\, q^{-2}$ vs. q^2 of an angle dependent PCS measurement in water from 30° to 130° at 15 °C, 35 °C and 55 °C.

As Figure S3 shows, except for very low scattering angles the diffusions coefficient D is constant for all q^2 values for all three temperatures.

Temperature dependent FTIR spectra

The following Figure shows a representative temperature dependent FTIR spectroscopy measurement.

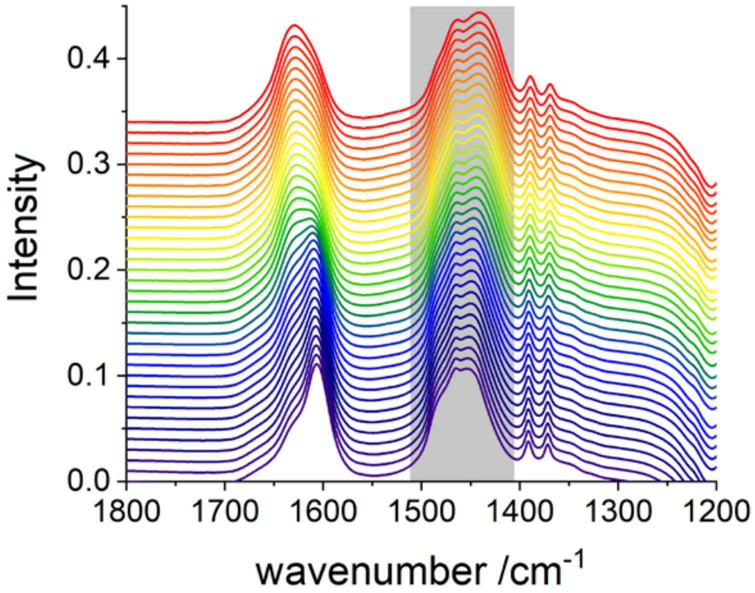

Figure S 4: FTIR measurement for a temperature range from 15.65 °C to 65.70 °C for a H-pNIPMAM microgel. The NH-band is highlighted in grey.
